# Longitudinal Study of Individual Exercises in Elite Rhythmic Gymnastics

**DOI:** 10.3389/fpsyg.2019.01496

**Published:** 2019-06-28

**Authors:** Elena Sierra-Palmeiro, Marta Bobo-Arce, Alexandra Pérez-Ferreirós, María A. Fernández-Villarino

**Affiliations:** ^1^Department of Physical Education and Sports, Faculty of Sports Science and Physical Education, University of A Coruña, A Coruña, Spain; ^2^Department of Especial Teaching, Faculty of Education and Sports Sciences, University of Vigo, Pontevedra, Spain

**Keywords:** code of points, apparatus, technical elements, performance analysis, rule change, world championship

## Abstract

The performance evolution in rhythmic gymnastics depends on changes in code of points. At the beginning of each Olympic cycle the code of points changes and therefore, the content of the competition exercises, as well. This study aimed to analyze – for each apparatus – the evolution of number of technical elements and final score over the last two decades (last 13 world championships), how they have been affected by changed code of points, and how the final score relates to the number of technical elements performed. The sample consisted of 416 exercises in five apparatus: ball (96), rope (40), hoop (96), ribbon (88), and clubs (96). The following variables were gathered: code of points, apparatus, technical group, total number of elements, final classification, and final score. Linear mixed-effects models were used to examine the effects on the number of elements and final score in each apparatus. The number of technical elements increased in all apparatus, between 7.4 and 20% over a 10-year period. There were mixed evolutions of final score between the different apparatus, between -6.3 and 14% over a 10-year period. There is small increase in number of elements in hoop and a small decrease in rope after a code change. There was a small decrease in final score in championships after a code change in hoop, moderate in clubs and ribbon, and large in rope. There was a negative relationship between number of elements performed and final score in clubs. In conclusion, the code change generally effects the final score negatively, but there were apparatus specific effects of code change on number of elements and relationship between number of elements and final score.

## Introduction

Rhythmic gymnastics is a sport that combines technical, aesthetic, and artistic parameters with the aim of reproducing an optimal execution model, both in matter of form and execution (Díaz- Pereira et al., 2014). When a gymnast performs her competition routine, she coordinates her body movements with handling of an apparatus (ball, clubs, tape, hoop or rope) in a choreographic composition accompanied by music.

The optimal execution model that the gymnast strive for is determined by both quantitative and qualitative criteria specified in the code of points. The code of points is set by the International Gymnastics Federation and are updated each Olympic cycle. It is therefore, considered the basis for the internal logic of the sport or strategy (the possibilities of interaction of gymnast with space, time, apparatus, with other gymnast, and with the criteria of success or failure), as well as a key factor when composing the competition routines and practice planning (Ávila-Carvalho et al., 2012a; Massidda and Calò, 2012; Leandro et al., 2017).

In the last 20 years, the International Gymnastics Federation has made numerous changes to the code of points aiming to increase the objectivity of the judges scoring, and to stimulate the development of the sport. These code changes have resulted in structural changes to the competition routines. For example, Ávila-Carvalho et al. (2011) have shown that the compositions of the routines have evolved toward increasing the difficulty and variety in body movements, and toward a higher technical mastery and enrichment in the apparatus handling.

In an attempt to understand the competitive model and performance in the rhythmic gymnastics, several studies have analyzed the composition of competition routines, both in group and individual events (Ávila-Carvalho et al., 2011, 2012a; Trifunov and Slovodanka, 2013; Agopyan, 2014). However, most have analyzed a single competition, and none have considered the changes in the code of points over time. Moreover, they have focused solely on the artistic content or on the difficulty of the body movements. Further, the results of these studies have highlighted the constant and rapid evolution of the sport, stressing the need to continuously perform these kind of studies (Čuk et al., 2012; Hökelmann et al., 2012; Massidda and Calò, 2012; Bučar et al., 2013; Pelin, 2013).

Given the limitations in earlier studies, there is a need for longitudinal studies analyzing the evolution of competition routine compositions and performance over time, which would provide valuable new information on the factors affecting the performance in rhythmic gymnastics (Ávila-Carvalho et al., 2012b; Leandro et al., 2017). Further, it could provide new insight into the effects of changes to the code of points on the performance. Finally, it would provide valuable insights for rhythmic gymnastics coaches, enabling them to make more informed decisions when planning practices and learning activities to improve the skills in each apparatus. This study aims to analyze – for each apparatus – the evolution of number of technical elements and final score over the last two decades (last 13 world championships), how they have been affected by changed code of points, and how the final score relates to the number of technical elements performed.

## Materials and Methods

### Sample

The sample consisted of 416 individual rhythmic gymnastics routines performed in the final round of each apparatus in the last 13 rhythmic gymnastics world championships (1997–2018). The total sample comprised 96 ball, 96 clubs, 96 hoop, 88 ribbon, and 40 rope routines. Only four of the apparatus are used in each competition, with the International Gymnastics Federation choosing which are to be included before each championship. Which apparatus were used in each championship is presented in Table 1. Eight routines were performed in all final rounds for each apparatus and championship. The study was conducted in accordance with the ethical standards of sports science (Harris and Atkinson, 2009). The study did not require an ethical committee approval, as only publicly available data was used.

**Table 1:** Code change, maximum score, and apparatus used in each championship.

Championship	Code change	Maximum score	Ball	Clubs	Hoop	Ribbon	Rope
Berlin 1997	Change	10	✓	–	✓	✓	✓
Madrid 2001	Change	30	✓	✓	✓	–	✓
Budapest 2003	Stable	30	✓	✓	✓	✓	–
Baku 2005	Change	20	✓	✓	✓	✓	–
Patras 2007	Stable	20	–	✓	✓	✓	✓
Mie 2009	Change	30	✓	✓	–	✓	✓
Moscow 2010	Stable	30	✓	✓	✓	–	✓
Montpelier 2011	Stable	30	✓	✓	✓	✓	–
Kiev 2013	Change	20	✓	✓	✓	✓	–
Izmir 2014	Stable	20	✓	✓	✓	✓	–
Stuttgart 2015	Stable	20	✓	✓	✓	✓	–
Pesaro 2017	Change	20	✓	✓	✓	✓	–
Sofia 2018	Stable	20	✓	✓	✓	✓	–


### Procedure and Variables

Video recordings of all routines were obtained from the International Gymnastics Federation website^1^. All recordings were checked to ensure that the entire surface of the floor and the gymnast’s performance were visible throughout the routine. Two international rhythmic gymnastics judges (Ph.D. in sports science and rhythmic gymnastics) with at least of 15 years of international judging experience observed the recordings and counted the number of elements performed in each routine. The final score was taken from the official records on the International Gymnastics Federation website. The year, apparatus, code of points used, number of elements, and final score was registered for each routine.

Each apparatus has a number of technical groups determined by the code of points (Table 2). The judges recorded the elements performed within each technical group of the apparatus used for the routine. The sum of elements in all technical groups was then considered as the total number of elements in the routine. To assess the reliability, 10 randomly chosen routines (two for each apparatus) were observed by both judges. After a period of 2 weeks, the routines were re-observed. The inter-rater reliability was, for ball ICC(2,1) = 1, clubs ICC(2,1) = 0.98, hoop ICC(2,1) = 1, ribbon ICC(2,1) = 0.96, rope = 0.87. The intra-rater reliability was, for ball ICC(2,1) = 0.96, clubs ICC(2,1) = 1, hoop ICC(2,1) = 1, ribbon ICC(2,1) = 0.96, rope = 0.87.

**Table 2:** Technical groups in each apparatus.

Apparatus	Technical groups
Ball	Throws and catches, bounces, roll on the floor, roll over body segments, unstable balance, and handling.
Clubs	Small circles, mills, throws and catches, tappings, asymmetric movements, unstable balance, and handling.
Hoop	Roll on the floor, roll over body segments, rotations, throws and catches, passing through, passing over, unstable balance, and handling.
Ribbon	Spirals, snakes, throws and catches, boomerang throw, releases, passing through, passing over, and handling.
Rope	Passing through with jump, passing through with a part of body, throws and catches, releases and catches, rotations, unstable balance, and handling.


The code of points was transformed into a dichotomous variable (code change), indicating whether the code of points used was the same as in the previous championship or had changed. No change of code of points was considered as reference in all analyses. As the maximal obtainable score varied between the different code of points, all scores were rescaled to a maximum of 20 points, which is the maximum score. To measure the evolution over time, a continuous variable indicating the year of the championship was used. The maximum point and whether the code of points changed for each championship is presented in Table 1.

### Statistical Analysis

All statistical analyses and graphics were produced using R 3.5.1. The mean and standard deviation (SD) for each apparatus and championship are presented graphically together with the occurrence of code change.

Linear mixed-effects models were used to assess the evolution over time and the effects of code change on the number of elements performed. A linear model was fitted to predict the number of elements for each apparatus, including year as continuous, and code change as dichotomous fixed effects. The models included random intercepts for the individual gymnasts, as well as for each competition. Further, linear mixed-effects models were also used to assess the evolution over time, effect of code change and of number of elements on the final score. A linear model was fitted to predict the final score for each apparatus, including year as continuous, code change as dichotomous, and number of elements as continuous fixed effects. The models included random intercepts for the individual gymnasts, as well as for each competition.

By using regression modeling, it is possible to assess the specific effects of each variable, accounting for the influence of the other ones. As each apparatus comprise its own independent competition, a model was fitted to each of them in both cases. To account for repeated measures of the same gymnast in several championships, gymnast was included as a random factor in the model. To account for the potential influence of different maximum scores in the championships on final score and number of elements, it was included as a fixed factor.

Initially, all two-way interactions were included in the model. However, the interaction between year and number of elements had no effect on the final score and was therefore excluded from the final models. The assumptions of normality and homoscedasticity of residuals were checked by inspecting qq-plots, plots of residual vs. predicted values, residuals vs. predictors and SDs for the different levels of the predictors. The inspection did not reveal violations of the assumptions.

The difference in number of elements and final score between championships with and without code change are presented using estimated marginal means, with 95% confidence intervals (CI). The evolution of number of elements and final score over a 10-year time period is presented by multiplying the simple effect of year by 10 and are also expressed as percentages of the grand mean. This is done both overall for the full sample and comparing between championships with and without code change. Finally, the relationship between final score and number of elements are presented as standardized effect sizes by multiplying the simple effects of number of elements by two SD of the number of elements in the original sample. Standardized effect sizes were also calculated by dividing the difference by the SD derived from all random variance components of the models (Westfall et al., 2014). The standardized effect sized for the number of elements are interpreted as small (≥0.2), moderate (≥0.6), large (≥1.2), very large (≥2.0) and extremely large (≥4.0) (Hopkins et al., 2009). For final score, the standardized effect sizes are interpreted as small (≥0.3), moderate (≥0.9), large (≥1.6), very large (≥2.5) and extremely large (≥4.0) (Malcata and Hopkins, 2014).

## Results

**FIGURE 1:**
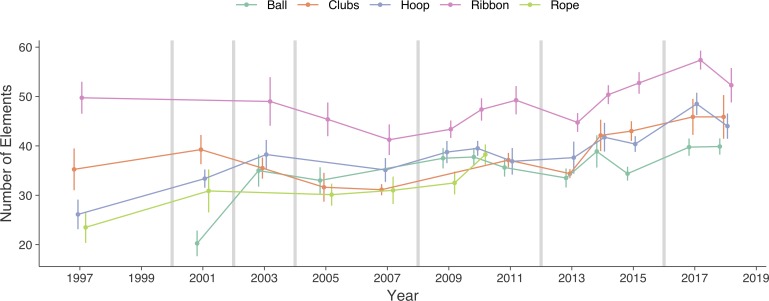
Evolution of average number of elements with standard deviation for each apparatus. Vertical lines indicate code change.

**FIGURE 2:**
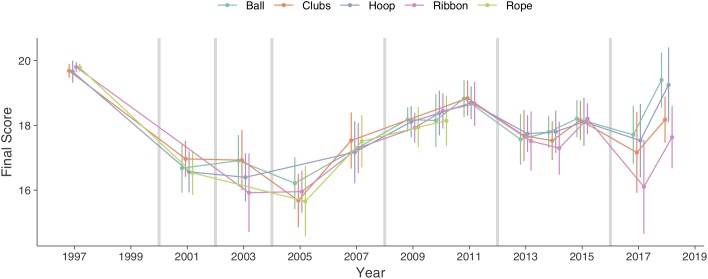
Evolution of average final score with standard deviation for each apparatus. Vertical lines indicate code change.

The mean and SD of number of elements and final score for each championship and apparatus are presented in Figure 1, 2. The evolution over time, together with indications of when code changes occurred are presented graphically for number of elements (Figure 1) and final score (Figure 2).

The estimated means of number of elements and final score for championships with and without code change, when accounting for change over time and number of elements, are presented in Table 3 together with the raw and standardized difference. There is no clear general difference in number of elements between championships with and without code change. There is small increase in number of elements in hoop and a small decrease in rope after a code change. The final score is generally lower in championships after a code change, with small difference in hoop, moderate in clubs and ribbon, and large in rope.

**Table 3:** Estimated means with and without code change for number of elements and final score.

	No code change			Code change								
						
		95% CI			95% CI			95% CI			95% CI	
								
	Estimate	Lower	Upper	Estimate	Lower	Upper	Difference	Lower	Upper	ES	Lower	Upper
***Number of elements***												
Ball	35.8	27.0	44.5	35.3	30.1	40.6	-0.4	-11.1	10.2	-0.1	-1.6	1.4
Clubs	38.3	32.8	43.8	37.9	32.2	43.6	-0.4	-8.7	7.8	0.0	-1.0	0.9
Hoop	37.4	34.9	39.9	39.7	36.8	42.6	2.3	-1.7	6.4	0.4	-0.3	1.1
Ribbon	48.7	44.3	53.0	48.4	43.3	53.6	-0.3	-7.1	6.6	0.0	-0.9	0.9
Rope	32.0	16.2	47.7	30.1	14.8	45.4	-1.9	-28.1	24.4	-0.3	-5.0	4.3
***Final score***												
Ball	17.5	17.0	18.1	17.5	17.2	17.9	0.0	-0.7	0.7	0.0	-0.5	0.6
Clubs	18.6	17.7	19.4	16.9	16.0	17.8	-1.7	-3.0	-0.4	-0.9	-1.7	-0.2
Hoop	18.3	17.5	19.2	17.4	16.5	18.4	-0.9	-2.2	0.4	-0.5	-1.2	0.2
Ribbon	18.2	17.8	18.6	16.6	16.1	17.0	-1.6	-2.2	-1.0	-1.1	-1.5	-0.7
Rope	19.0	16.1	21.8	16.1	13.6	18.6	-2.9	-9.0	3.2	-2.1	-6.6	2.4


*CI, confidence interval; ES, effect size.*

The estimated evolution over a 10-year period of number of elements and final score, when accounting for all other effects, are presented in Table 4. Moreover, the estimated evolution for championships with and without code change, as well as difference are also presented in Table 4. The number of elements has increased for all apparatus. Over a 10-year period, it increased 20% in ball, 12% in clubs, 20% in hoop, 7.4% in ribbon and 13% in rope. The final score seems to have increased in ball and decreased in clubs and rope. Over a 10-year period, the change was 14% in ball, -1.7% in clubs, 2.8% in hoop, -0.6% in ribbon and -6.3% in rope. The increase is higher in ribbon and rope.

**Table 4:** Estimated 10-year evolution overall, with and without code change for number of elements and final score.

				No code change			Code change								
							
		95% CI			95% CI			95% CI			95% CI			95% CI	
										
	Estimate	Lower	Upper	Estimate	Lower	Upper	Estimate	Lower	Upper	Difference	Lower	Upper	ES	Lower	Upper
***Number of elements***															
Ball	7.1	-7.9	22.1	5.4	-18.8	29.6	8.8	-1.6	19.3	3.4	-18.8	25.6	0.5	-2.7	3.6
Clubs	4.7	-1.0	10.5	5.9	-2.6	14.4	3.5	-6.9	14.0	-2.4	-17.6	12.9	-0.3	-2.0	1.5
Hoop	7.6	4.6	10.6	7.8	3.7	12.0	7.3	1.9	12.8	-0.5	-8.0	7.1	-0.1	-1.4	1.2
Ribbon	3.6	-2.3	9.6	3.1	-4.3	10.5	4.2	-6.3	14.6	1.0	-12.6	14.6	0.1	-1.7	1.9
Rope	4.1	-27.7	35.8	6.3	-39.5	52.2	1.8	-42.2	45.7	-4.6	-68.2	59.0	-0.8	-12.1	10.4
***Final score***															
Ball	2.5	1.6	3.5	3.2	1.7	4.7	1.9	1.1	2.6	-1.4	-2.8	0.1	-0.2	-0.4	0.0
Clubs	-0.3	-1.2	0.7	-1.2	-2.6	0.2	0.7	-1.0	2.4	1.8	-0.7	4.3	0.2	-0.1	0.5
Hoop	0.5	-0.6	1.5	-0.2	-1.6	1.2	1.1	-0.7	3.0	1.3	-1.2	3.9	0.2	-0.2	0.7
Ribbon	-0.1	-0.7	0.5	-1.4	-2.1	-0.7	1.2	0.3	2.2	2.7	1.4	4.0	0.4	0.2	0.5
Rope	-1.1	-6.1	4.0	-3.5	-8.8	1.7	1.4	-10.4	13.3	5.0	-4.5	14.5	0.9	-0.8	2.6


*CI, confidence interval; ES, effect size.*

The estimated standardized effect of number of elements on the final score, when accounting for all other effects, are presented in Table 5 together with the separate effects for championships with and without code change and the difference. The effects are reported as the amount of change in final score produced by a change of 2 SD in number of elements. There is no clear general relationship between number of elements performed and final score. The only clear relationship is a negative one in clubs. In ball there is a small difference, with a negative relationship in championships without rule change, and a positive relationship in championships with code change. In rope, there is also a small difference, with a positive relationship in championships without rule change, and a negative relationship in championships with code change.

**Table 5:** Estimated effect of two standard deviation change in number of elements on final score overall, with and without rule change.

				No code change			Code change								
							
		95% CI			95% CI			95% CI			95% CI			95% CI	
										
	Estimate	Lower	Upper	Estimate	Lower	Upper	Estimate	Lower	Upper	Estimate	Lower	Upper	ES	Lower	Upper
Ball	0.3	-0.4	1.1	-0.4	-1.6	0.8	1.1	0.1	2.0	1.5	0.0	3.0	0.3	0.0	0.5
Clubs	-0.6	-1.2	0.1	-0.3	-1.1	0.6	-0.9	-1.8	0.1	-0.6	-1.8	0.6	-0.1	-0.3	0.1
Hoop	0.0	-0.5	0.6	0.3	-0.7	1.3	-0.2	-0.8	0.4	-0.5	-1.7	0.6	-0.1	-0.2	0.1
Ribbon	0.2	-0.4	0.8	0.2	-0.6	1.0	0.2	-0.7	1.1	0.0	-1.1	1.2	0.0	-0.1	0.1
Rope	-0.1	-0.6	0.3	1.0	0.4	1.7	-1.3	-2.0	-0.7	-2.3	-3.2	-1.4	-0.3	-0.4	-0.2


*CI, confidence interval; ES, effect size.*

## Discussion

The aims of this study were to analyze the evolution of the performance in rhythmic gymnastics in the last two decades and describe how the number of technical elements performed with the apparatus are related to the performance of the gymnasts. In general, the changes to the code of points have affected the number of technical elements performed and the final score differently for the different apparatus. The relationship between number of technical elements and final score were also apparatus specific. According to Ávila-Carvalho et al. (2012b), the technical apparatus elements used in the routine composition varies according to the type of apparatus, stressing the importance of studying each apparatus separately. If the work with an apparatus implies a technique and a specific coordination with the body, it also implies specific motor and physical performance (Botti and Nascimento, 2011). However, care should be taken when interpreting the results for rope, as it has not been present in any world championship since 2010.

During the two decades observed, the number of technical elements has increased for all apparatus. This suggests that the impact of apparatus skills on the final score has increased, as well as the coordination between the movement of the apparatus and body (Tsopani et al., 2012). In the same way, this increase has meant that each modification to the code of points have sought an increased precision in the performance evaluation criteria. There was no clear effect of code change on the number of elements or its evolution over time, which might mean that the code changes had not substantial effect on the number of elements. It might also mean that the different code changes affected the number of elements differently. Leandro et al. (2015) points out that the updates to the code of points are directed toward increasing the complexity of the interaction between the gymnast and the apparatus in the routines, either through an increase in number of elements or the degree of coordination difficulty. The characteristics of the specific apparatus might affect the amount of possible ways for the gymnasts to interact with the apparatus.

In championships with a new code of points, the final score decreased. That is, a code change significantly affects the performance of the gymnasts, who must modify their workouts and learn new possible technical elements. Aspects such as the variety and diversity of the compositions can be compromised, and until a stabilization of the score code occurs, the richness and spectacularity of the compositions diminish. Similarly, a code change means both new evaluation criteria and implementations, as well as the fact that new technical elements are introduced. All this contributes to the quality of the artistic composition of a routine and to the development of the sport (Levre, 2011). Leandro et al. (2017) point out the difficulty of making an accurate judgment in the elements of the apparatus. Particularly, the technical elements of the apparatus, such as masters, are considered by the judges to be less objective to evaluate. The work with the apparatus sometimes demands an extraordinary coordination, perfect technical control and specificity that hinder the work of the judge. Each new code change requires a search by the coaches for skilful interaction (gymnast-apparatus) and the increase of technical elements of the apparatus in the compositions, with the aim of improving the performance in competition. In the same way, for the judges it requires the definition of new objective criteria that allows a correct and precise evaluation of the performance.

As for the relationship between number of elements and final score, the only clear findings were that higher number of elements relates to lower final score in clubs. As pointed out above, the specific characteristics of each apparatus affects the relationship between number of elements and final score. There seem to be a more positive relationship between number of elements and final score in competitions with rule change in ball and a negative one in rope. Probably, as with the hoop and clubs, the characteristics of the ball and the rope would allow us to understand this relationship. However, the lack of data related to rope does not allow us to have an objective assessment (it has not been in competition for the last 8 years).

The present study has some limitations that should be considered. The analysis of competition routines should also focus on qualitative aspects of the technical elements of each apparatus, such as variety and diversity. These aspects are determining factors in the quality and richness of the compositions and they contribute to the development and evolution of the sport, and therefore its modernization. Therefore, an evaluation of judges and coaches on the changes that the code of points makes, both in the training models and in the evaluation criteria, would increase the perspective of analysis of the presented data.

## Conclusion

Throughout the evaluated period, the number of technical elements of the apparatus that the gymnasts perform in their competition routines has increased, although each time the code of points change the performance of the gymnasts decreases. With each Olympic cycle new forms of coordination between gymnast and apparatus must be developed, which has increased the repercussion of the technical elements of the apparatus in the score of the competition routines and the complexity of the compositions.

This type of analysis provides information about the performance indicators as well as knowing how they are affected by the changes in the code of points. This information can be used by coaches to improve the composition strategies of the exercises, both increasing the complexity of the technical elements and the interaction between gymnast and apparatus. It also provides information to the judges for the definition of new evaluation criteria that allows a better adjustment of the evaluation in competition.

## Data Availability

The datasets generated for this study are available on request to the corresponding author.

## Author Contributions

All authors participated in the study design, documentation, development, and writing of the manuscript.

## Conflict of Interest Statement

The authors declare that the research was conducted in the absence of any commercial or financial relationships that could be construed as a potential conflict of interest.

## Footnotes

1 www.gymnastics.sport
